# Risk factor for children in the pandemic: the use of cleaning products at home

**DOI:** 10.3205/dgkh000451

**Published:** 2023-10-20

**Authors:** Emine Güdek Seferoglu, Ümran Çevik Güner

**Affiliations:** 1 Kütahya Health Science University, Faculty of Health Science, Kütahya, Turkey; 2 Gaziosmanpaşa University, Faculty of Health Science, Tokat, Turkey

**Keywords:** Child, Disinfectant, Mother, Pandemic, Safe use

## Abstract

**Background:**

Intensified cleaning protocols to maintain a safe environment during the pandemic caused an increase in the use of disinfectants. The use of cleaning products in safer conditions by mothers is one of the important practices that will reduce the risk of household accidents.

**Objective:**

The aim of research was determine the practices of mothers about the safe use of cleaning and disinfectant products in the COVID-19.

**Methods:**

Data were collected by online survey among 255 mothers of the children 0-6 age from April and July 2021. Percentage, mean and chi-square tests were used to evaluate the data.

**Results:**

It was reported that the amount of cleaning product usage (69%) increased significantly, 26.3% of the mothers store the products in a locked cabinet and 29.4% use the product in the recommended amount. It was detected 28.7% of the mothers use disinfectants close to children. It was detected that 37.6% of the families were exposed to cleaning and disinfectant products. There was not significant difference between exposure situations and maternal age, education, employment status.

**Conclusions:**

It can be suggested that health workers should organize screening and training programs for the community about safe cleaning and disinfection practices.

## Zusammenfassung

**Zielsetzung:**

Intensivierte Reinigungsprotokolle zur Aufrechterhaltung einer sicheren Umgebung während der Pandemie führten zu einem Anstieg des Einsatzes von Desinfektionsmitteln. Die Verwendung von Reinigungsmitteln unter sichereren Bedingungen durch Mütter ist eine der wichtigen Praktiken, die das Risiko von Haushaltsunfällen verringern. Das Ziel der Forschung bestand darin, die Praktiken von Müttern hinsichtlich der sicheren Verwendung von Reinigungs- und Desinfektionsprodukten bei der COVID-19-Erkrankung zu ermitteln.

**Methode:**

Die Daten wurden per Online-Umfrage unter 255 Müttern der Kinder im Alter von 0 bis 6 Jahren im April und Juli 2021 erhoben. Zur Auswertung der Daten wurden Prozent-, Mittelwert- und Chi-Quadrat-Tests verwendet.

**Ergebnisse:**

Es wurde berichtet, dass die Menge der verwendeten Reinigungsprodukte deutlich zunahm (69 %), 26,3 % der Mütter lagerten die Produkte in einem verschlossenen Schrank und 29,4 % verwendeten das Produkt in der empfohlenen Menge. Es wurde festgestellt, dass 28,7 % der Mütter Desinfektionsmittel in der Nähe ihrer Kinder verwenden. Es wurde festgestellt, dass 37,6 % der Familien Reinigungs- und Desinfektionsmitteln ausgesetzt waren. Es gab keinen signifikanten Unterschied zwischen den Expositionssituationen und dem Alter, der Bildung und dem Beschäftigungsstatus der Mutter.

**Schlussfolgerungen:**

Es kann vorgeschlagen werden, dass Gesundheitspersonal Screening- und Schulungsprogramme für die Gemeinde zu sicheren Reinigungs- und Desinfektionspraktiken organisieren sollte.

## Introduction

Since the home and surrounding environment are basic living area for the children between 0 and 6 years of age, home accidents are particularly important for this age group. Home accidents are ignored unless they cause an injury that requires intervention in children. However, frequent minor injuries may also address serious injuries. Majority of home accidents is preventable. Therefore, it is important to reduce the risk factors that lead to accidents in the home environment [1]. 

It was determined that the coronavirus disease (COVID-19) caused by the SARS-CoV-2 virus may be transmitted through droplets and by touching the mouth, nose and eyes after contact with contaminated surfaces [2], [3]. Proper and regular cleaning and frequent disinfecting of objects and surfaces in houses and community environments are recommended in order to reduce the risk of COVID-19 and other viral respiratory diseases [4]. Within this context, intensified cleaning protocols to maintain a safe environment during the pandemic caused an increase in the use of disinfectants [5], [6]. However, inadequate or excessive use of these products cause potential health risks for the users. A significant increase in calls to poison centers about exposure to cleaners and disinfectants has been reported since the onset of the COVID-19 pandemic. The Netherlands National Venome Information Center (NVIC) announced that disinfectant-induced poisoning events occurred approximately 5 times more than normal periods during the COVID-19 pandemic period, and poisoning cases among children between 0 and 4 years of age increased significantly. The National Poison Data System (NPDS) in the United States reported that the rate of calls for exposure to cleaners and disinfectants between January and March, 2020 increased by 20.4% when compared to the same period of the previous year. The NPDS data do not provide information showing a precise association between exposures and cleanup efforts during the COVID-19 pandemic; however, they do show a clear time relation with the increased use of cleaning and disinfectants [7]. The Canadian Poison Information Monitoring Center (CPIMC) reports that calls for selected cleaning and disinfectant products increased by 400% in 2020 when compared to the previous year, with the most calls (42%) related to the inappropriate use of bleach [8]. Li et al. found in their study that especially 3-year-old children, who have more hand contact and mouth movements, have consistently higher exposure than other age groups as a result of contact with disinfected surfaces [5]. The CDC has published a guideline with general recommendations for routine cleaning and disinfection of homes during the COVID-19 [4]. Limited knowledge exists about the use or abuse of cleaning products during the COVID-19 pandemic [8]. The use of cleaning products in safer conditions by mothers is one of the important practices that will reduce the risk of household accidents (i.e. poisoning, burns). Nurses are healthcare professionals who can advise on the adequate use of cleaning and disinfection products and the frequency of cleaning and disinfection at home in order to protect the health of children, families and the community. Accordingly, it is important for nurses to determine the practices of women in the safe use and storage of cleaning and disinfectant products at home and to correct their faulty practices. There is limited number of studies about this subject, and the association with the pediatric health was not investigated. The research was planned to determine the practices of mothers of the children between 0 and 6 years of age about the safe use of cleaning and disinfectant products in the home environment during the COVID-19.

## Materials and methods

### Design 

This is a cross-sectional and descriptive study. 

### Sample 

The universe of the study consisted of mothers of the children between 0 and 6 years of age living in different regions of Turkey, and the sample of the study consisted of 255 mothers who volunteered to participate in the study and whose questionnaires were sent online using the snowball sampling method. It was calculated in the power analysis that 234 mothers could be included when the effect size was taken as 0.19 according to the accident rates seen in children in the literature [9], [10] the significance level was taken as 0.05, and the power size was taken as 0.85. The inclusion criteria include having a child between 0 and 6 years of age, being literate, and accepting to participate into the study. Mothers who have youngest children at and above 7 years of age were excluded. 

### Measures 

The question form for mothers’ use of home cleaning and disinfectant products during COVID-19 pandemic were used in the research.

*The Question Form for Mothers’ Use Of Home Cleaning And Disinfectant Products During COVID-19 Pandemic*: This form which was prepared by the researchers in line with the literature [4] consists of 40 questions including five questions about the socio-demographic characteristics of the participants such as age, education, employment status, number of children, 35 questions including information sources during the COVID-19 pandemic, exposure situations, and the use of cleaning and disinfectant products at home. 

### Data Collection 

The data of the study were collected between April and July 2021. The data collection form was transferred to the online environment through Google Forms and then the link (URL) required to access the form was sent to the people who met the criteria determined for the participants. The purpose and scope of the research were explained, the information that the rights and identities of the participants would be protected by keeping them confidential was shared, the instruction regarding the implementation process was presented, and it was stated that they could contact the researcher for questions or problems they might encounter during the application. The research complied with the Declaration of Helsinki and permission was obtained from the Ethics Committee of a university (No. 2021/04-13) for the research. Online consent was obtained from the parents with an information document stating the nature of the study, the confidentiality issues, and the voluntary participation. 

### Data Analysis 

The data coding and evaluation was done in the computer environment with the SPSS 22 package program. Percentage, mean and chi-square tests were used to evaluate the data. Any p value below 0.05 (p<0.05) was accepted as statistically significant. 

## Results

Demographic characteristics of the mothers was presented in Table 1 (Tab. 1). It was determined that 45.5% of the mothers participating in the study were between 31 and 40 years of age, 75.8% were university graduates, 61.7% were employed, and 80% had only one child. 

**Figure 1:**
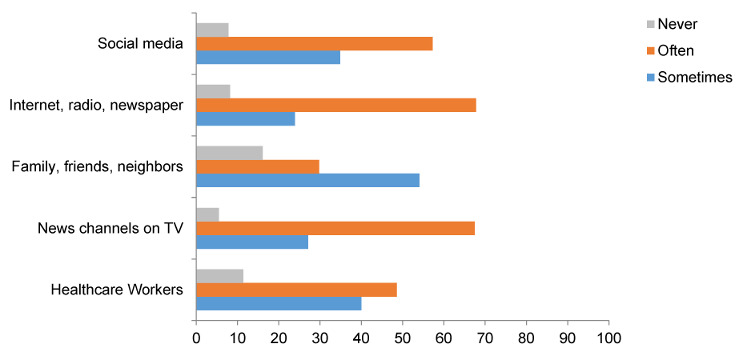
The information resources of the mothers about cleaning products

The information resources of the mothers about cleaning products are provided in Figure 1 (Fig. 1). It is seen that 67.8% of the mothers obtained information through internet, radio, newspaper, 67.5% of them were informed through television, 57.3% through social media, and 48.6% through health workers. 

The practices of mothers about storage of cleaning products at home are given in Table 2 (Tab. 2). While 87.5% of the mothers commonly store their cleaning products in the bathroom and 71.0% in the kitchen, only a little more than a quarter (26.3%) store the products in a locked cabinet, almost all mothers prefer cleaning products that are almost all sold in closed packages (99.2%), keep such products away from foods (98.0%), store these products by closing the caps (97.3%); 9.8% of them stated that they filled the cleaning products in other containers, only a little more than half (57.6%) usually ventilate the area where they store their cleaning products. 

Cleaning and disinfection practices of mothers during the pandemic are given in Table 3 (Tab. 3). Among the mothers, 65.1% stated that there was an increase in the frequency of cleaning and disinfection when compared to the pre-pandemic period, 56.5% stated that they perform cleaning 2 to 3 times a week and 19.2% clean every day; 69.0% stated that there was an increase in the amount of cleaning products used. Furthermore, two questions were asked about the quantity of the cleaning products used over the bleach sample, 64.7% of them said that they added bleach to every five liters of water while cleaning surfaces touched frequently; however, 58.0% of them said that they added bleach to each liter of water while cleaning the toilet and bathroom with random quantity. 

**Figure 2:**
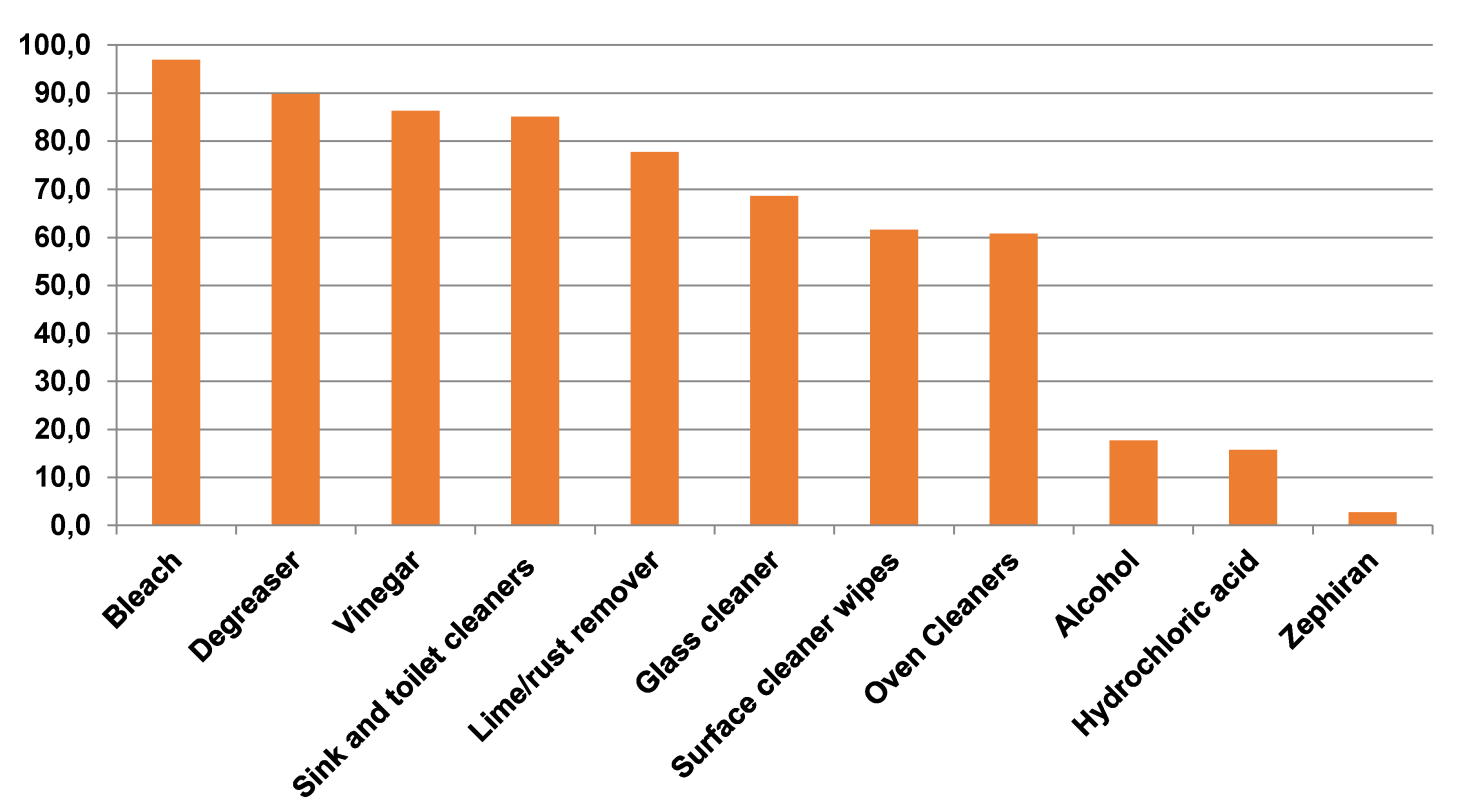
The cleaning and disinfectant products that are commonly used at home during the pandemic

The cleaning and disinfectant products that are commonly used at home during the pandemic are given in Figure 2 (Fig. 2). It was determined that the most common products were bleach (96.9%), degreaser (89.8%), vinegar (86.3%), and sink and toilet cleaners (85.1%). 

**Figure 3:**
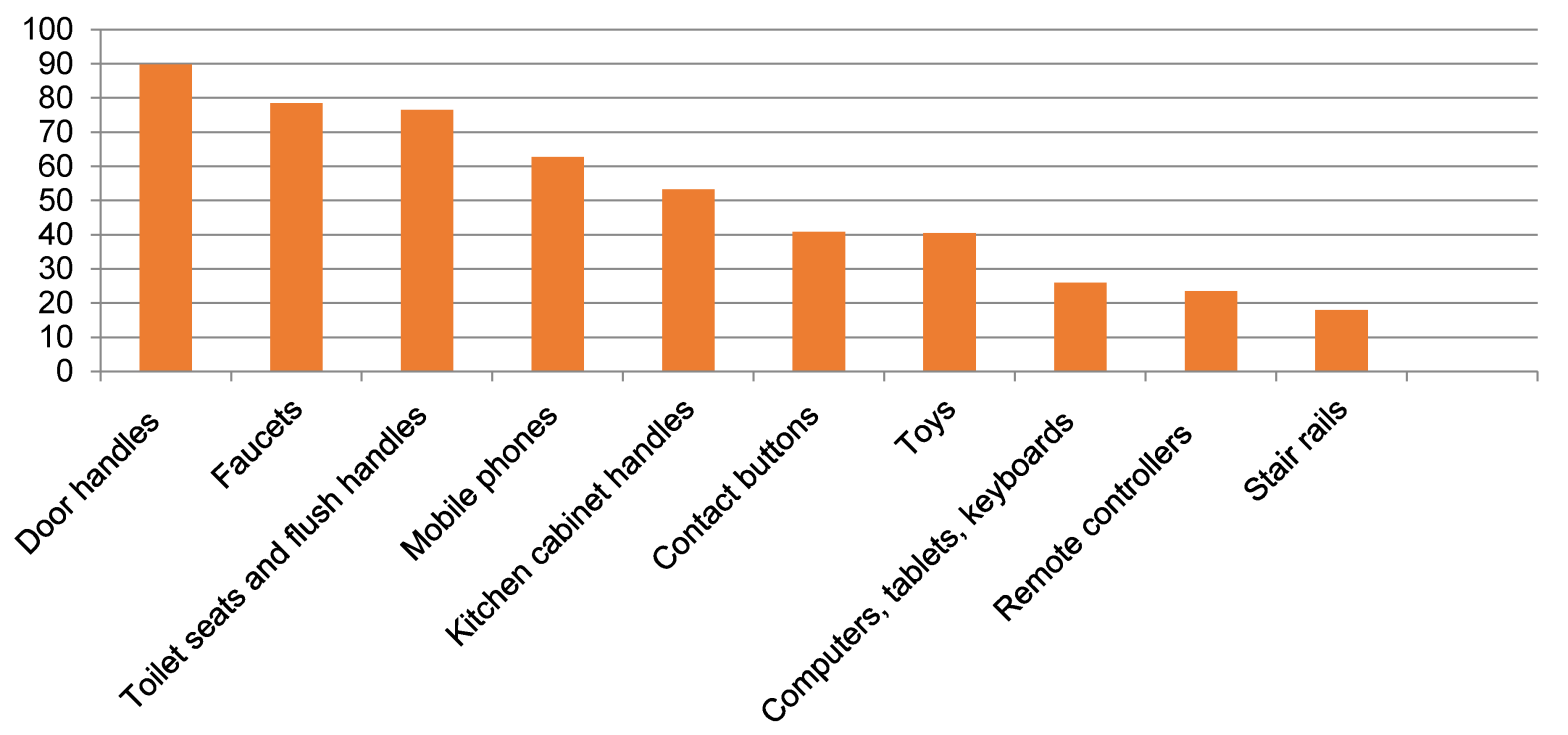
The surfaces that are commonly cleaned at home during the pandemic

The surfaces that are commonly cleaned at home during the pandemic are provided in Figure 3 (Fig. 3). Mothers stated that the most frequently cleaned surfaces were door handles (89.8%), faucets (78.4%), toilet seats, flush handles (76.5%) and mobile phones (62.7%).

The safe use of cleaning and disinfectant products by mothers during the pandemic is provided in Table 4 (Tab. 4). It was detected that only 38.4% of the mothers read the instructions for use on the cleaning and disinfectant product, 59.6% ventilate the environment before and after using the cleaning and disinfectant product, 29.4% use the product in the recommended amount, 21.2% wear gloves during the procedure, and 2.0% use goggles. Mothers expressed that they use cleaning and disinfectant products by mixing with each other during cleaning (sometimes by 23.5%; always by 5.9%), they use hot water (sometimes by 41.2%; always by 12.5%), 40.0% while preparing the disinfected water, they leave the disinfectant on the surface for at least one minute, mix the bleach with vinegar ammonia or another cleaning product (sometimes by 16.5%; always by 6.7%), 46.7% of them do not clean the dirty surfaces with detergent before disinfecting, and 31% stated that they do not use the prepared bleach within the recommended time. Furthermore, 28.7% of the mothers stated that they use disinfectants close to their children, and 44.7% stated that it is not necessary to remove the asthmatic child or adult from the environment during cleaning. In this study, 38.8% of the mothers used these products for hand and skin cleaning, 5.1% used them for mouthwash, and 8.6% used them for food cleaning such as adding bleach to the washing water of fruits and vegetables.

The situation of exposure to cleaning and disinfectant products at home during the pandemic is given in Table 5 (Tab. 5). Exposure to cleaning and disinfection products at home during the pandemic is 37.6%. It was also observed with the results that 80.2% of the mothers were exposed; however, one of every 8 children (0 to 2 years: 5.2%; 3 to 6 years: 7.3%) is exposed. It has been determined that approximately half of the exposure (48.9%) is caused by skin contact, and 36.5% by inhalation of the product.

The association between exposure to cleaning and disinfectant products and sociodemographic characteristics of the mothers is given in Table 6 (Tab. 6). There was not any statistically significant difference between exposure situations and maternal age, education, employment status and the number of living children. 

## Discussion

The present study was conducted to determine the practices of mothers of the children between 0 and 6 years of age about the safe use of cleaning and disinfectant products in the home environment during the COVID-19 pandemic. It was reported in the studies on referrals to pediatric emergency services in our country that the most common poisonings were caused by caustic/corrosive substances, and most of these cases were caused by products used in household cleaning [10], [11], [12]. Since household cleaning products are not stored in safe conditions, this plays an important role in child poisoning cases. It was detected in our study that most of the mothers keep the cleaning and disinfectant products in the kitchen and bathroom, at a reachable place by the children, unprotected or on the ground, uncompliant storage conditions for safety; however, limited number of mothers keep them in locked cabinets. The reason for this behavior of mothers may be that they want to access these products more easily while cleaning. Furthermore, almost half of the mothers reported that they do not ventilate the areas where they store their products. The review of the studies on the storage conditions of cleaning products at home revealed that women have similar behaviors [13], [14], [15], [16], [17], [18]. Since almost all of the mothers in our study reported that they keep cleaning products away from food is a positive finding in reducing the contact of products with food; however, the preference of keeping these products in the kitchen facilitates the access of children and suggests that mothers do not have sufficient knowledge about the behaviors leading to the exposure.

Cleaning products should be stored in their own packages. In this study, 9.8% of the mothers stated that they transferred their cleaning products to different containers such as bottles and glasses. However, this may cause mixing of cleaning products with drinks and food, and poisoning of small children in particular. Silva et al. [16] and Gollu et al. [18] respectively reported in their studies that 12% and 31.3% of the participants do not store cleaning products in original containers. It may be suggested in consideration of the studies that this inaccurate practice still continues among women with a decrease when compared to previous years [13], [14], [15], [16], [17], [18], [19].

The fast transmission of the pandemic and causing death increase the health concerns of individuals, cause them to store cleaning materials and take more precautions regarding hygiene, cleaning and contact [20]. The CDC recommends that disinfection is not necessary; however, frequently touched surfaces are cleaned and disinfected every few days, unless there is a sick or positive individual at home for COVID-19 in the last 24 hours [3]. In this study, 65.1% of the mothers reported that they increased the frequency of cleaning and disinfection at home during the COVID-19 pandemic. However, mothers stated that they also increased the amount of cleaning and disinfection products used during this process. Findings of the studies conducted by Koksoy Vayisoglu & Oncu [21], Gharpura et al. [22], and Zheng et al. [6] are in line with our findings. Inaccurate, unnecessary or excessive use of cleaning agents and disinfectants may cause damage for human health due to skin, eye and respiratory tract problems, family economy and the ecosystem due to the increase in the concentration of these chemicals in wastewater [23], [24], [25]. 

The CDC states that disinfection of objects and surfaces that are commonly touched after cleaning is important to reduce the risk of COVID-19. In our study, almost half of the mothers stated that they did not clean the dirty surfaces with a detergent before disinfection, and this indicates that they did not perform a proper disinfection process. Furthermore, since more than half of the mothers do not consider electronic items such as toys, electric switches, keyboards, remote controls which are frequently used during the pandemic as surfaces that need to be cleaned and disinfected frequently, this may cause virus transmission through these objects. Similarly, it was detected in a study conducted in Iran during the pandemic period that most of the participants have usually disinfected surfaces such as mobile phones, kitchen, bathroom, and toilet; however, this rate was limited for computers [25]. 

It was detected in our study that approximately two of every five households were exposed to cleaning and disinfectant products during the pandemic, and mothers as well as their children at and below six years of age were most frequently exposed in these households. It was reported that exposure is most common by skin contact and inhalation of the product, less frequent by ingestion and eye contact. Koksoy Vayisoglu & Oncu [21] reported in their study on adults that the prevalence of health problems related to the use of cleaning products during the pandemic was 47%. It was stated in the same study that the most frequently reported problems were skin problems and shortness of breath. The use of cleaning and disinfectant products has increased to reduce the risk of infection in homes along with the onset of the COVID-19 pandemic. This has caused an increase in exposure rates. The Canadian Poison Center data suggests that calls about exposure to hand and household disinfectants in March 2020 were 4-times more when compared to the same period of the previous year, and nearly half of the calls were associated with the bleach [8].

It was determined in our study that maternal age, education, employment status and the number of children living at home did not affect the exposure. However, it is stated in the literature that exposure to corrosive substances as a home accident is more common in societies with lower education levels. Gollu et al. [18] stated that as the education level of mothers increases, they store corrosive substances under adequate conditions and have the accurate knowledge and attitude about corrosive substance use; however, this is not affected by maternal age. Ucuncu et al. [26] also found that mothers with a higher level of education who work and have less than three children have more knowledge about home accidents, and the age of the mother does not affect the level of knowledge. In this study, most of the mothers who work could not work outside because it was compulsory to stay at home during the pandemic period. It may be considered in this respect that the employment status of the mother is not effective on exposure. However, although the majority of the mothers were university graduates, the fact that these levels of education did not have an effect on avoidance of exposure suggests that all mothers tended to different hazardous practices in order to protect and avoid the disease due to panic and fear as the threat perception posed by the pandemic increased.

Behaviors such as use of cleaning products more than recommended on the label, mixing different products are stated as hazardous behaviors. Mixing bleach with vinegar or ammonia releases chlorine and chloramine gases that may cause severe damage on the lung tissue [24]. The use of the bleach with hot water also causes appearance of this gas. The CDC recommends using water at room temperature for dilution of disinfectants unless otherwise stated on the product label, the cleaning solution including bleach within 24 hours, and leaving the disinfectant applied on the surfaces for at least one minute [4]. Assessment of our study findings in line with these recommendations of the CDC revealed that almost half of the mothers used hot water while preparing disinfectant water, and one-third mixed different disinfectants with each other. Such attitude of mothers reveals the risk of poisoning by inhalation. It was detected in our study that the mothers prepare the bleach to be used for disinfection randomly rather than accurate concentration and do not use it within the recommended time, and the disinfectant do not contact with the surface for an adequate time. These results indicate that mothers have limited knowledge about the safe preparation of cleaning and disinfectant solutions and they cannot perform an effective disinfection process. Previous studies reported that women mix different detergents during house cleaning, use more detergents than necessary and take minimum precautions [27], and 74% of the bleach is prepared with an inaccurate concentration during the pandemic period [25]. 

CDC recommends reading the instructions for use prior to the cleaning and disinfection, using protective equipment such as gloves and goggles, and ventilating the environment during the procedure [4]. The use of disinfectants may trigger asthma attacks in children with asthma. Therefore, it should be ensured that the child should be kept in another room while cleaning [28], [29]. It was reported that babies living in homes where cleaning products are used commonly are at higher risk of problems such as wheezing and asthma when they reach the age of three [30]. It was found in our study that most of the mothers do not follow the recommendations of CDC, mothers do not read the instructions for use on the cleaning and disinfection product, do not use the product with the recommended amount, do not wear gloves and glasses during the process, and do not ventilate the environment regularly. Furthermore, since the number of mothers who reported that disinfectant products could be used while an asthmatic child or adult is present in the environment is higher, this suggests that these mothers have insufficient knowledge about the use of cleaning and disinfectant products and have hazardous practices that would affect their health negatively, and this situation has an important role for higher exposure. Similarly, Garcia-Hidalgo et al. conducted a study in Switzerland reported that only half of the participants wear gloves when using bleach, this rate was 22% for toilet cleaner and 1% to 11% for other cleaning products [30]. On the other hand, Dindarloo et al. stated that 16.5% of the participants wear glasses during the disinfection of surfaces; however, 47.5% do not use any personal protective equipment during the pandemic [25]. In the study conducted by Gharpure et al. during the Covid pandemic, 71% of the participants stated that it is necessary to wear gloves and 64% of the participants stated that wearing glasses is necessary as personal protective equipment during the use of cleaning and disinfection products at home [22]. Silva et al. [16] reported that 55% of the participants and Göllü et al. [18] 37% of the participants do not read the labels of disinfectant products they use at home. It was detected in the study of Koksoy Vayisoglu & Öncü that half of the participants used ventilation constantly during the cleaning process; however, limited number of these use protective equipment regularly [21]. As previous studies in the literature indicate, individuals show lower compliance in reading the labels of the products and using protective equipment against the harmful effects of disinfectants both before and after pandemic. 

Another hazardous behavior is the use of household cleaning products in inadequate areas. In this study, almost of the mothers used these products for hand and skin cleaning; however, a limited number of them used such products for food cleaning purposes such as gargling or adding bleach to the irrigation water of fruits and vegetables. Similarly, the previous studies conducted during the pandemic reported that these practices which have a higher risk are carried out in homes [21], [22].

## Limitations

The limitations of the present study include the inability to conduct a personal survey with parents and their children, since the research was conducted in a certain time period and the pandemic continued at the time of the study. Furthermore, another limitation is that only parents with online access were able to participate in the study. Therefore, it is recommended to make arrangements to include mothers/fathers with no/limited internet access in future studies. 

## Conclusion and suggestions

It was detected that the knowledge of the mothers on the safe use of cleaning and disinfectant products is insufficient and they are involved in hazardous practices that will adversely affect the health of themselves, their children and other family members. Safe cleaning and disinfection practices protect the health of the children and reduce the risk of exposure, not only during the pandemic, but at all times. Accordingly, it may be suggested that healthcare workers, especially nurses, should organize training programs by using innovative strategies (web-based education, social media) and scanning the whole society about safe cleaning and disinfection practices. It is recommended that nurses working in pediatrics plan regular training on parents of children between 0 and 6 years of age about protection from home accidents and raising awareness.

## Notes

### Competing interests

The authors declare that they have no competing interests.

### Acknowledgments

We thank the mothers who participated in this study.

### Annotation

This study was presented as a oral presentation at the “International 6^th^ Forensic Nursing Congress” (16–18 May 2022) 

### Ethical considerations

Approval was obtained from the Ethics Committee of the Institute of Health Sciences of a University (No. 2021/04-13).

### Financial Resource

This study was not financially supported. 

### Authors’ ORCIDs:

- Emine Güdek Seferoglu: 0000-0001-5803-0059
- Ümran Çevik Güner: 0000-0002-8677-0414

## Figures and Tables

**Table 1:**
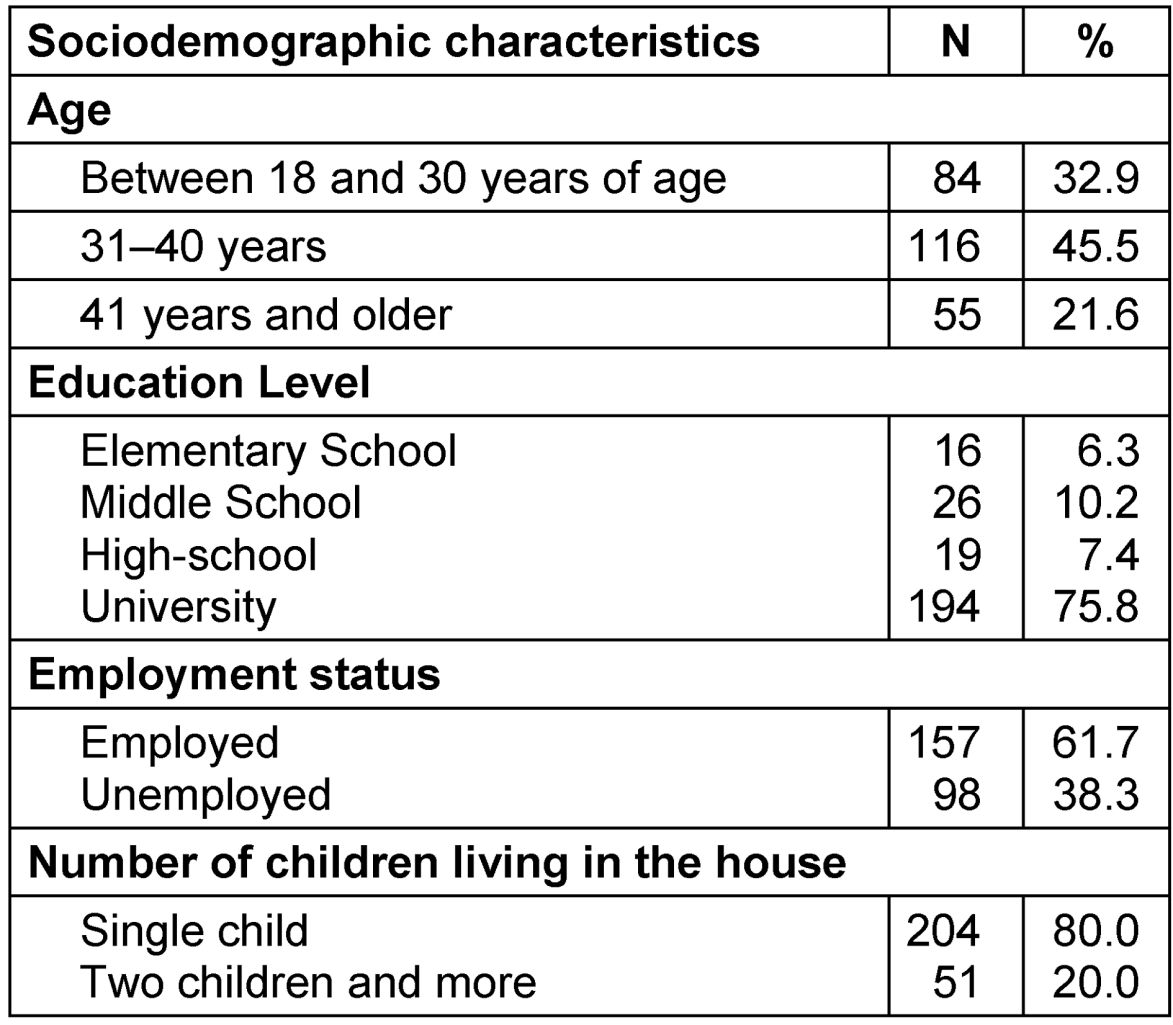
Sociodemographic characteristics of mothers

**Table 2:**
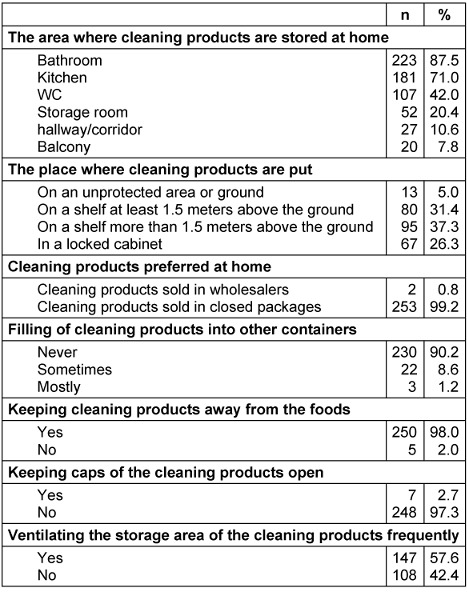
The practices about storage of cleaning products at home

**Table 3:**
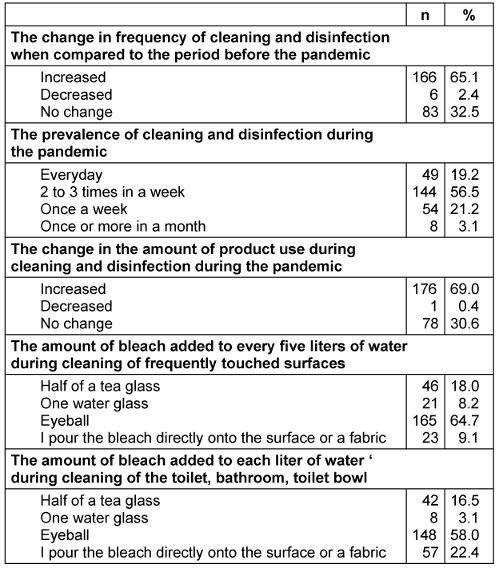
Cleaning and disinfection practices of mothers during the pandemic

**Table 4:**
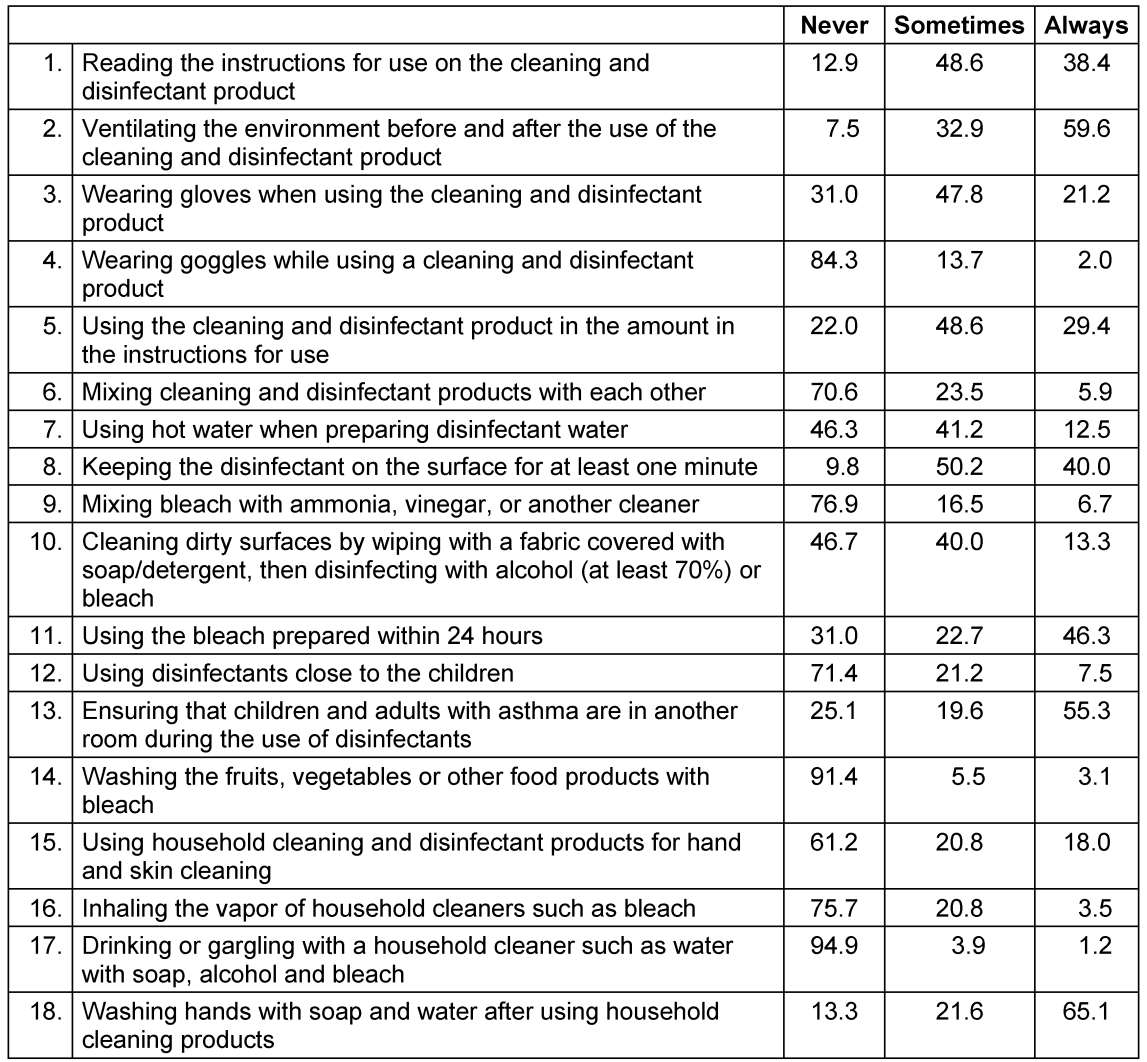
Safe use of home cleaning and disinfectant products

**Table 5:**
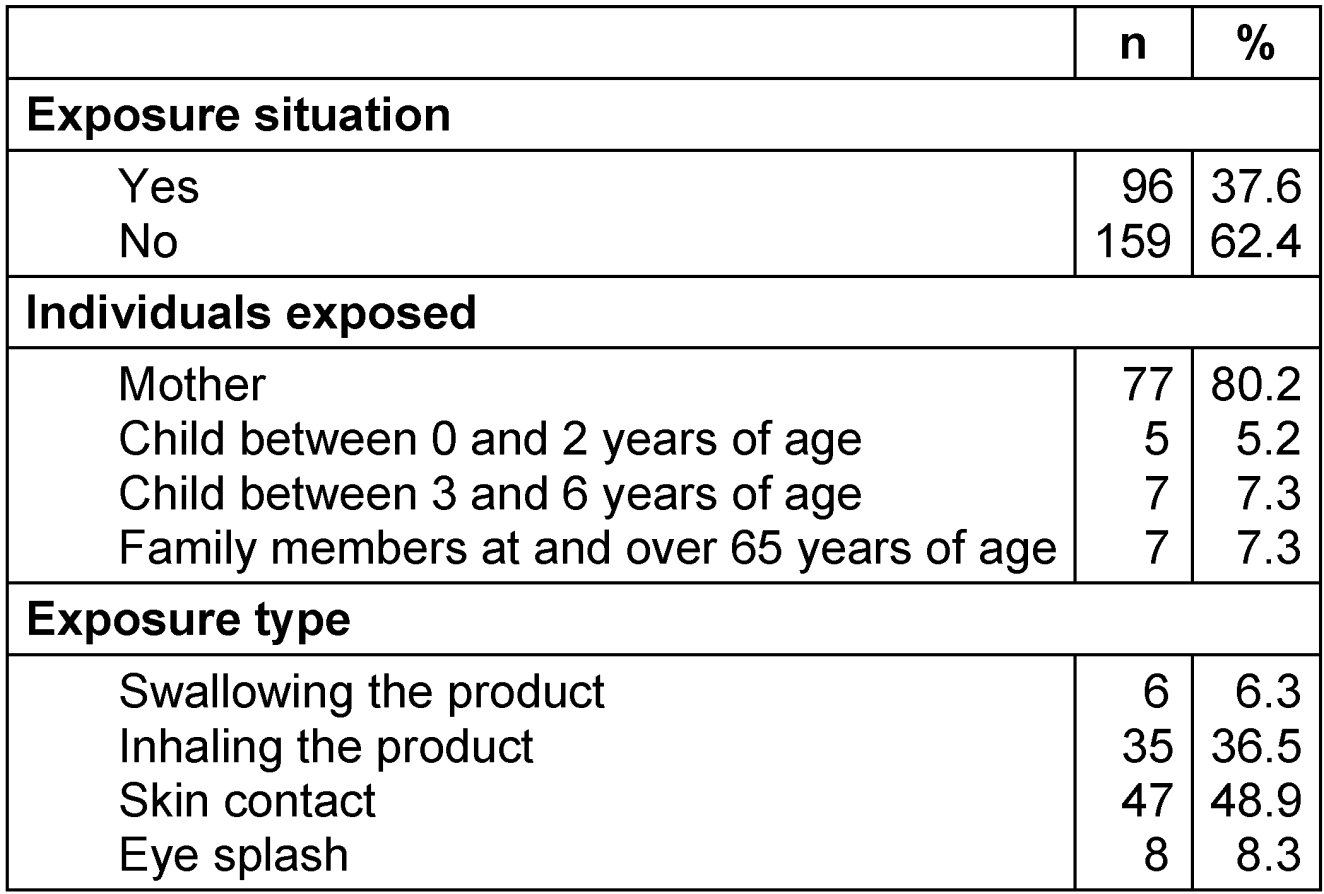
The situation of exposure to cleaning and disinfectant products at home during the pandemic

**Table 6:**
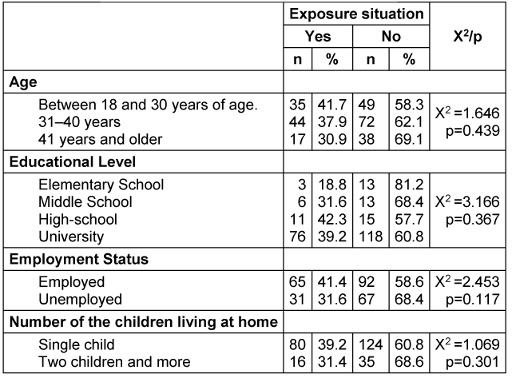
The exposure situation of mothers to cleaning and disinfectant products
